# Molecular tumor board availability and organization in sarcoma referral centers in Italy: are we on the right track?

**DOI:** 10.3389/fonc.2026.1641281

**Published:** 2026-06-03

**Authors:** Concetta Elisa Onesti, Beatrice Casini, Annarosaria De Chiara, Valentina Fausti, Roberto Luksch, Giuseppe Maria Milano, Emanuela Palmerini, Sabrina Vari, Serena Ceddia, Federica Riva, Toni Ibrahim, Renato Covello, Gennaro Ciliberto, Katia Scotlandi, Virginia Ferraresi

**Affiliations:** 1 Sarcomas and Rare Tumors Departmental Unit, IRCCS Regina Elena National Cancer Institute, Rome, Italy; 2 Pathology Unit, IRCCS Regina Elena National Cancer Institute, Rome, Italy; 3 Pathology Unit, Istituto Nazionale Tumori-IRCCS-Fondazione ‘G. Pascale’, Naples, Italy; 4 Advanced cellular therapies and rare tumors, IRCCS Istituto Romagnolo per lo Studio dei Tumori (IRST) “Dino Amadori”, Meldola, Italy; 5 Pediatric Oncology Unit and Biostatistics for Clinical Research Unit, Fondazione IRCCS Istituto Nazionale dei Tumori, Milan, Italy; 6 Division of Pediatric Hematology and Oncology, Gene and Cellular Therapy, IRCCS Ospedale Pediatrico Bambino Gesù, Rome, Italy; 7 Osteoncology, Bone and Soft Tissue Sarcomas and Innovative Therapies Unit, IRCCS Istituto Ortopedico Rizzoli, Bologna, Italy; 8 Scientific Direction, IRCCS Regina Elena National Cancer Institute, Rome, Italy; 9 Experimental Oncology Laboratory, IRCCS Istituto Ortopedico Rizzoli, Bologna, Italy

**Keywords:** molecular medicine, MTB, referral center, sarcomas, survey

## Abstract

**Background:**

With the rapid progress of molecular medicine, new target therapies for solid tumors have become available, leading to the creation of molecular tumor boards (MTBs) designated to evaluate potential individualized treatment options. Unlike other solid or liquid tumors, there is no standard and customized tool to analyze sarcoma. We analyzed the organization of MTB in referral centers for sarcomas treatment in Italy.

**Materials and methods:**

A 30-question survey was designed in 2021 by the Regina Elena National Cancer Institute and distributed among Italian referral centers for pediatric and adult sarcomas. This survey was developed as part of Alliance Against Cancer (Alleanza Contro il Cancro, ACC) project to provide a descriptive analysis of the availability and organization of MTBs as well as the propensity to offer genomic profiling to sarcoma patients.

**Results:**

A total of 6 out of 10 centers contacted answered the survey, and all stating centers declared to have an MTB. The composition of MTB was variable, with a dedicated oncologist in 83% of centers, although all cases discussed required the presence of the oncologist. 83% of centers met the ACC criteria for eligibility at MTB discussion. In 83% of cases both the primary and the metastases were analyzed, while in 17% only the metastasis were analyzed. The type of analysis available were target panel sequencing and whole exome sequencing (WES) in 100% of centers, whole genome sequencing (WGS) in 83%, RNA sequencing in 53%. Bioinformatics analysis software used were Illumina pipeline in 50% of centers; Ion Reporter (Thermo Fisher Scientific), QIAGEN CLC Genomics Variant Reporter and Archer Analysis in 33%, 17% and 33% of centers respectively. The knowledge base software for variant interpretation in precision oncology were Oncomine Reporter and Sophia, both in 17%. Other specific tools (e.g., CibersortX, GATK suite, and DEseq2) were used in 33% of centers. The time required for the analysis was ≤ 10 days in 33% of centers and > 10 days in 67%. In 83% of centers results are stored locally, and a database with clinical data and follow-up was recorded.

**Conclusion:**

MTBs were present in most sarcoma referral centers that answered the survey, albeit with different organizational arrangements. Although this survey should be regarded merely as a descriptive analysis of the early stages of MTB use in sarcomas, and is not representative of the national landscape as a whole, it highlights the clinical need to develop expertise in using MTB for rare cancers and to standardize the process and may serve as a basis for future larger-scale, prospective efforts aimed at harmonizing MTB practices across Italian sarcoma centers.

## Introduction

With the advent and increasing widespread use of molecular medicine, new treatment opportunities for cancers are emerging. In particular, for neoplasms in which standard therapies are no longer effective, the possibility of seeking new therapeutic options through genomic studies is becoming increasingly common in the field of oncology. The role of molecular medicine for diagnosis and treatment choice is better known in some malignancies, such as non-small cell lung cancer (NSCLC), breast, ovarian, and colorectal tumors (1–5). For cancer such as NSCLCs, the Food and Drug Administration (FDA) has recommended the use of comprehensive genomic profiling (CGP) rather than the use of single genomic assays, given the high number of currently known molecular targets (2). However, for most cancers, including rare ones, the use of molecular medicine to search for new therapeutic targets remains an option usually confined to the time of exhaustion of standard therapeutic options. A multidisciplinary approach based on the foundation of Molecular Tumor Boards (MTBs), was for the first time described by the Michigan University in 2011, and then rapidly grew (6, 7). MTBs are generally composed of several professional figures such as medical oncologist, radiation oncologist, surgeon, radiologist, pathologist, geneticist, molecular biologist, bioinformatician, pharmacist, basic/translational scientist, immunology expert, and research/clinical trial coordinator (7, 8). The presence of pathologists and of molecular biologists, is critical to choose the appropriate technique to be used among the various sequencing modalities (e.g., whole-exome sequencing (WES), whole-genome sequencing (WGS), RNA sequencing, Target sequencing with CGP, and circulating tumor DNA sequencing (ctDNA) (7, 8). In addition, the analysis of the large amount of data requires the presence of a bioinformatician. Also, the possible therapeutic indications must be evaluated, with the support of a pharmacist (7).

In Italy, the Alliance Against Cancer (Alleanza Contro il Cancro, ACC) drafted guidelines on MTB. The ACC is an Italian cancer research network founded by the Ministry of Health. Its mission is to transfer the results of basic research into clinical practice and to promote technological and organizational innovation in the fight against cancer. The ACC comprises cancer research institutes and general hospitals with a research focus (9).

Recently, classifications to rate molecular alterations based on clinical evidence as therapeutic targets have been implemented. Among these, one of the most widely used in Europe is the European Society for Medical Oncology Scale for Clinical Actionability of molecular Targets (ESMO/ESCAT), which identifies 6 levels of evidence for the molecular targets examined: I ready for routine use, II still investigational and in need of further data, III clinical benefit demonstrated in other cancers or in similar molecular alteration, IV evidence available only at the preclinical level, V evidence implying co-targeting, and X no evidence of actionability (10). Another similar 5-tier classification system is the OncoKB, which is based on FDA and National Comprehensive Cancer Network (NCCN) guidelines, expert panel recommendations, and literature data (11–13). Finally, a classification system from the Association for Molecular Pathology/American Society of Clinical Oncology/College of American Pathologists (AMP/ASCO/CAP) is also used, based on 4-tiers to categorize somatic variations based on their significance: Tier I variants with strong clinical significance, Tier II variants with potential clinical significance, Tier III variants of unknown clinical significance, and Tier IV variants deemed benign or likely benign (14).

Sarcomas are rare tumors, with an incidence of less than 6 cases per 100, 000 inhabitants per year, which can affect soft tissue or bone, with approximately 80 known histotypes (15). Despite major advances in the treatment of almost all solid tumors thanks to new immunotherapy strategies and molecularly targeted therapies often guided by a molecular profilation, chemotherapy is still the therapeutic mainstay of sarcomas. This is because of several factors, such as the biological characteristics of these tumors, the heterogeneity of histological subtypes, and the difficulty in conducting randomized clinical trials, due to the rarity of the disease and sometimes lack of pediatric clinical trials. In addition, the age of onset, often in the young population, results in patients having a longer life expectancy and consequently often exhausting currently available standard therapeutic lines when they are still in good general clinical condition.

Currently, the use of next generation sequencing (NGS) in sarcomas is recommended by ESMO at diagnosis (16). This is due to the extreme heterogeneity of sarcoma histologic subtypes and frequent misdiagnosis using routine histopathologic techniques such as hematoxylin-eosin staining and immunohistochemistry (16–20). Furthermore, NGS can facilitate the selection of molecularly driven therapies in sarcoma, aiming to identify new potential treatment opportunities (19).

To assess the level of interest in genomic profiling of sarcomas, an online survey was administered in 2021 to Italian referral centers for pediatric and adult sarcomas as part of a project of ACC. All of the participating centers were also members of the Italian Sarcoma Group (ISG), a nonprofit association of clinicians and scientists involved in sarcoma research. The purpose of this article is to analyze the diffusion and organization of MTB for sarcoma care by presenting the results of the survey and to show an update until today of published articles on the use of MTB in the real-life setting, in order to evaluate the evolution of this kind of approach.

## Materials and methods

The survey was designed by an expert medical oncology (VF) and an expert molecular biology (BC) from the Sarcoma Disease Multidisciplinary Team (DMT) at the coordinating center, the Istituto di Ricovero e Cura a Carattere Scientifico (IRCCS) National Cancer Institute Regina Elena (IRE) in Rome. The IRE is a referral center for sarcomas of the ISG and the European Reference Network on Rare Adult Cancers (EURACAN) (21). Prior to submission to the centers, the contents of the survey were shared with the ACC coordinator of the Muscoloskeletal Tumors (MSKT) Working Group at the IRCCS Istituti Ortopedici Rizzoli (IOR) of Bologna, another ISG and EURACAN referral center. Ten sarcoma referral centers in Italy from the MSKT Working Group of ACC were invited to answer the survey in March 2021 by the on-line Microsoft Office Forms platform (22). The medical oncologist responsible for sarcomas was deemed the professional representative of each center; this person was sent the questionnaire and was responsible for ensuring that the data provided was reflective of their center.

The survey consisted of 30 questions: 4 questions regarding the data of the compiling center and healthcare professional; 21 multiple-choice questions; and 5 open-ended questions (**Table 1**).

**Table 1:** Online survey.

Experience of MTB in individual center
1. Center 2. Name Professional 3. E-mail 4. Telephone number 5. How many new cases/year of sarcoma are evaluated by your center? 6. How many cases/year of sarcoma patients are evaluated for discution by a MTB (internal or external to the facility)? 7. Does your center have a Molecular Tumor Board? 8. What professionals are included? 9. Are there any other criteria your center uses for MTB eligibility in addition to those listed? 10. If YES to question 9, indicate the other criteria 11. Given the rarity of Sarcomas and the limited therapeutic options, when is MTB proposed? 12. The case is proposed for evaluation by: 13. Is the patient given an identification code and is it recorded on an IT platform? 14. Informed consent is required for the presentation of the case to the MTB (explaining the meaning of the proposed assessment), for the collection, conservation and use of the biological material and the data associated with it for the purpose of carrying out biomolecular investigations for research and of potential therapeutic interest:
About molecular analysis discussion on MTB
15. The proposed molecular analysis of the tumor during the MTB is preferentially performed on: 16. What technologies are available at the center? Specify the massively parallel sequencing platforms in use. 17. What kind of advanced sequencing analyses can be proposed? 18. What bioinformatics analysis software are used? List 19. Do you use the support of bioinformaticians? 20. Is there a local data storage system? 21. Genomic profiling is performed: 22. How are detected genetic alterations found? Levels of evidence: 23. How long does it take to ensure sample analysis?
About MTB decision
24. When discussing the molecular data, is the MTB's collegial decision reported in a report? 25. After the molecular tests are performed, who reports the results? 26. Who communicates the MTB suggestions to the patient? 27. In the event of a molecular result predictive of response to an off-label drug and in the event of the impossibility of accessing nominal/compassionate use programs or clinical trials, what is the procedure used for accessing the drug? 28. If you answered OTHER to question 27, please enter any comments. 29. Does the center participate in national or international clinical trials of targeted therapy guided by genomic profiling? 30 Does a computer database exsist with the follow up of the discussed patients managed by the MTB?

The survey investigated the following issues: the number of new cases and cases evaluated by the MTB per year; the composition of the MTB; the eligibility criteria used to discuss a patient at MTB and the time at which the molecular evaluation is proposed according to ACC criteria (**Table 2**); the sampling, molecular and bioinformatics techniques used; the criteria for evaluating the levels of evidence used; the time to the receipt of results; the way the data were stored; the communication of results to the patient; and the way experimental drugs were prescribed (9).

**Table 2:** ACC criteria.

Eligibility criteria for MTB
1. Exhaustion of standard therapeutic lines according to specific disease indications (metastatic disease or locally advanced inoperable) 2. Tumor with resistance to available standard treatments 3. Clinical/preclinical evidence of potential relevance of targets not routinely assessed 4. Rare pathologies or particular histologies with limited therapeutic options 5. Orphan tumors for which no appropriate treatment is available 6. Presence of incidental biomolecular data from previous analyses indicating actionable molecular targets suggested by scientific evidence 7. Unusual clinical history suggesting the execution of a molecular profile with potential therapeutic relevance 8. Family history suggestive of hereditary mutation (BRCA, MSH) to identify potential therapeutic targets, prognostic factors, preventive strategies for patient and family 9. Age ≥ 18 years 10. Life expectancy ≥12 weeks 11. Performance status ECOG ≤1

The results obtained were presented as a percentage of responses over the total number of participants and presented graphically with bar charts.

## Results

From March to May 2021, six of the ten invited centers filled out the survey. Five of these were national cancer institutes (IRCCS IRE of Roma, IRCCS IOR of Bologna, IRCCS Istituto Nazionale Tumori of Milan, IRCCS “G. Pascale” Foundation of Napoli, IRCCS Istituto Romagnolo per lo Studio dei Tumori “Dino Amadori” of Meldola) and one was a children’s hospital (IRCCS Ospedale Pediatrico Bambino Gesù of Rome). Of these centers, 3 had a number of new cases of bone (BS) or soft tissue sarcomas (STS) per year > 200, one between 180 and 200, the remaining 2 centers had a number of new cases ≤ 50. In detail, the number of new cases reported per year from each institution in ascending order were: 30, 50, 180–200, 240, 250–270, and 450.

All 6 responding centers had a MTB, that evaluated less than 10 sarcoma cases per year in 3 centers, and more than 10 cases in the remaining 3 centers. On average, only 14 cases were discussed at each MTB meeting, ranging from 2 to 30 cases. Specifically, the number of cases evaluated for discussion by MTB from each institution in ascending order were: 2, 5, 8, 15–20. 20, and 30.

**Figure 1:**
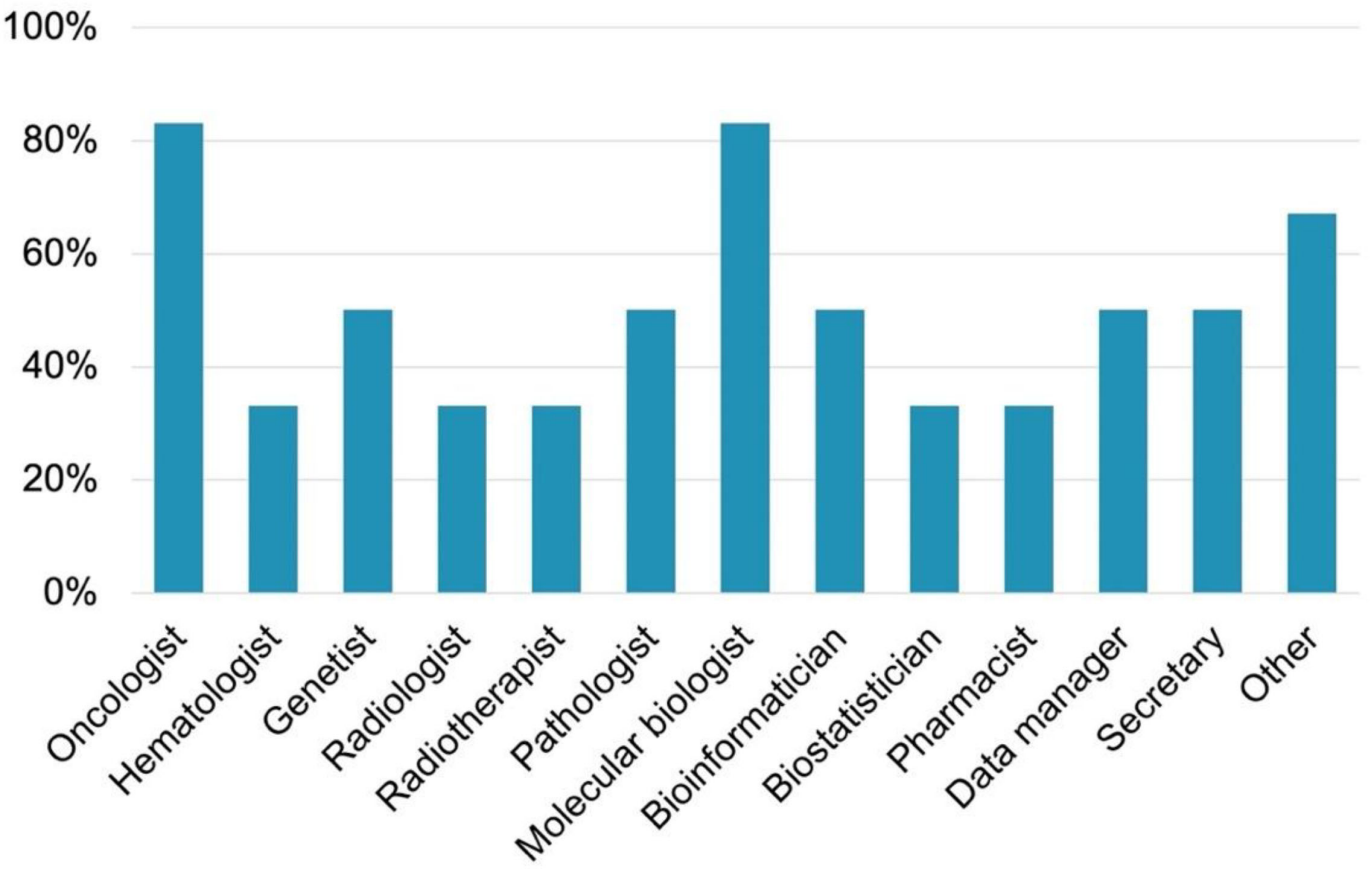
Healthcare professionals’ composition of the MTB.

The composition of the MTB was variable (**Figure 1**), with a dedicated oncologist present in 5 of 6 centers (83%), a geneticist in 3 (50%), a hematologist, a radiation oncologist, and a radiologist in 2 (33%). A molecular biologist was present in 5 centers (83%), and a pathologist in 3 (50%). A bioinformatician and a biostatistician were present in 3 (50%) and 2 centers (33%), respectively. A hospital pharmacist was present in 2 centers (33%), a data manager and a secretary in 3 (50%).

Regarding the criteria used for MTB eligibility, most centers used ACC criteria (5 out of 6, 83%), while 1 center stated they evaluated the individual patient on a case-by-case basis (17%).

In 4 centers (67%) evaluation at MTB was proposed at exhaustion of standard treatment options, in 1 center (17%) at diagnosis and at exhaustion of standard treatment, while 1 center (17%) stated that it proposed MTB evaluation after the first line of therapy.

In all 6 centers, cases were proposed for evaluation at the MTB by the treating oncologist, in 4 cases after discussion at the DMT meeting.

The specimens analyzed consisted of only metastasis tissue in 1 center (17%) and both primary tumor tissue and metastases in the remaining 5 centers (83%).

Genomic profiling was carried out in all cases internally; 2 centers were also supported from a private institution, and one from another external research center. The time required to get the results was ≤ 10 days in 2 centers (33%) and > 10 days in the remaining 4 (67%).

**Figure 2:**
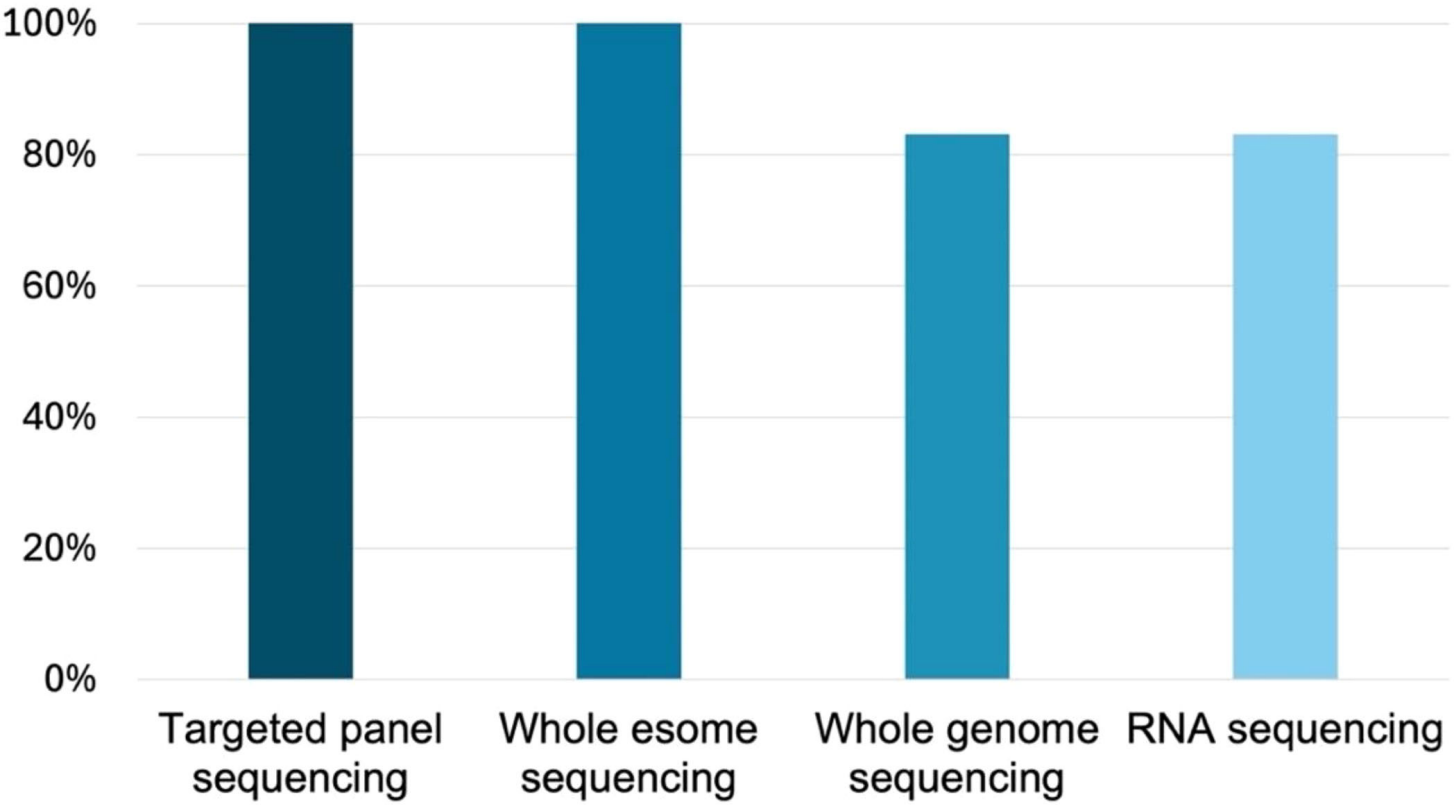
Biomolecular techniques available in the interviewed centers.

The sequencing platform used were Illumina in 5 cases (83%), Thermofisher in 3 (50%), and other in 2 (type not specified in the survey). The type of analysis available (**Figure 2**) were target NGS sequencing, mainly by CGP panel, in 6 centers (100%), WES in 6 (100%), WGS in 5 (83%), RNA sequencing in 5 (53%).

**Figure 3:**
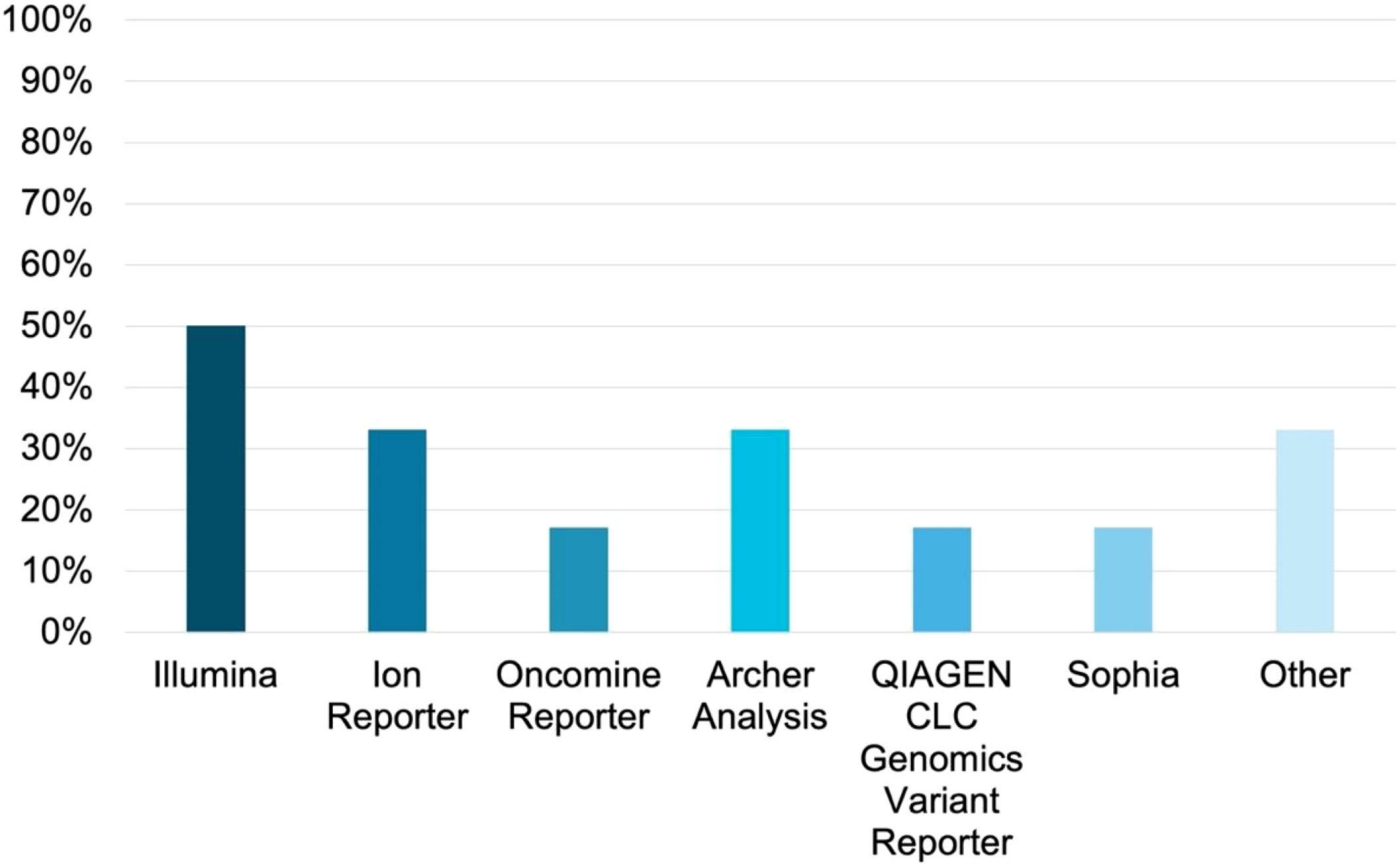
Bioinformatics analysis software used in the interviewed centers.

Of the 6 centers surveyed, 5 were supported by a bioinformatician for data analysis. Bioinformatics analysis software used (**Figure 3**) were Illumina in 3 centers (50%), Ion Reporter in 2 (33%), Archer Analysis in 2 (33%), Oncomine Reporter in 1 (17%), QIAGEN CLC Genomics Variant Reporter in 1 (17%), Sophia in 1 (17%), and other specific ones (e.g., CibersortX, GATK suite, and DEseq2) in 2 centers (33%).

**Figure 4:**
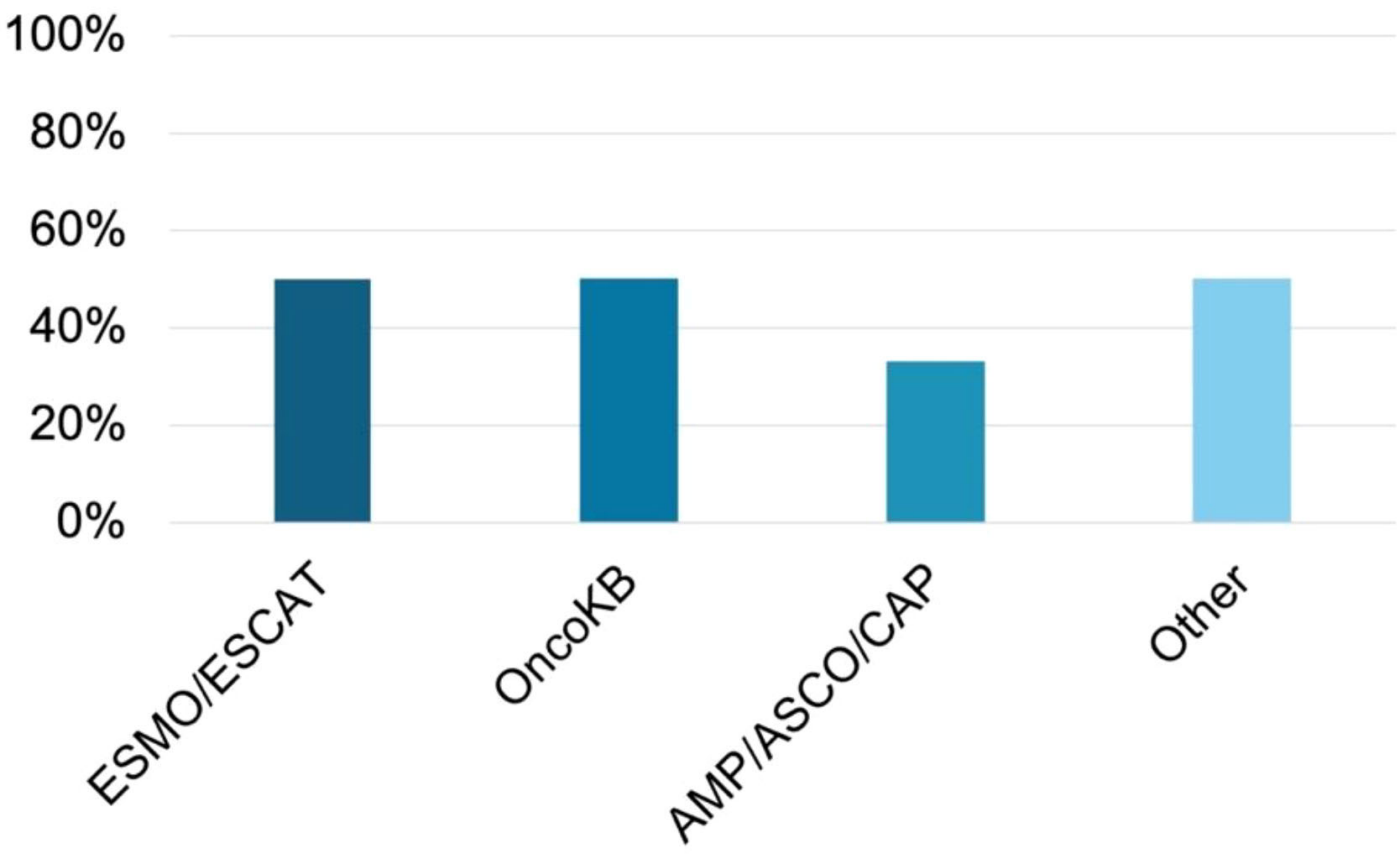
Level of evidence criteria used by the interviewed centers.

The level of evidence (**Figure 4**) of identified molecular alterations were evaluated according to ESMO/ESCAT criteria in 3 centers (50%), according to OncoKB criteria in 3 (50%), according to AMP/ASCO/CAP criteria in 2 (33%), and according to others unspecified criteria in 3 centers (50%) (10–14).

The results obtained were discussed at the MTB meeting by both oncologists and molecular biologist in 4 centers (67%), only by oncologists in 1 center (17%), and only by molecular biologists in 1 (17%). The collegial decision of the MTB was recorded in a record by 5 of 6 centers (83%). Results were always communicated to the patient by the treating oncologist (100% of centers).

In the case of a molecular result predictive of response to an off-label drug, in the absence of clinical trials or nominal/compassionate use programs, drug costs were taken over by the individual institution in all the centers (100%); in 4 of them (67%), specific programs funded by the National Health Service, such as the AIFA fund 5%, were also used (23).

Of the centers surveyed, 4 (67%) were participating in genomic profiling clinical trials at a national level and 5 centers (83%) at an international level.

In 2 centers (33%), patients signed specific consent for molecular analysis, while in the remaining 4 (67%), it was not required, as it is part of the general informed consent for data collection, storage and use of biological material including genetic data, administered at the patient’s first hospital admission. In 5 out of 6 centers (83%), patients were given an identification code, results were stored locally, and a database with clinical data and follow-up was recorded.

Two of the five open questions (numbers 10 and 28) did not receive an answer.

## Discussion

The growing interest in genomic profiling-based medical approaches and the flourishing of MTB has led to the need to regulate their activities. In Italy, a 2021 decree law proposed by National Agency for Regional Health Services (AGENAS) established the criteria, methods, and procedures for the establishment of MTBs within regional oncology networks and for the identification of centers specialized in performing NGS testing (article 8, paragraph 1- bis of Decree-Law No. 152 of 2021). This decree is also tasked with defining the competencies and rules of operation of MTBs, as well as how and when to collect data on the results of NGS genomic profiling tests performed by these specialized centers.

Currently, the Italian context is variegated, and even in sarcoma reference centers, the criteria for selecting patients for genomic profiling, both in terms of specific sarcoma histologic subtypes and disease setting, vary greatly, ranging from centers that offer this option to all patients (generally in research institutes with internal MTB) to centers that propose genomic profiling when standard therapeutic options are exhausted, to approaches that are more tailored to individual patients and often evaluated only by the medical oncologist. In terms of personalized medicine in Italy, the drugs currently approved for use and reimbursable (ESCAT score IA/B) in sarcomas are tyrosine kinases inhibitors (TKIs) such as imatinib, sunitinib, regorafenib, ripretinib, and avapritinib in GISTs, imatinib in dermatofibrosarcoma protuberans, and pazopanib in various histological subtypes of soft tissue sarcomas, without demonstration of the target (23–34). It is also possible to use TRK inhibitors (larotrectinib, entrectinib) approved in the presence of NTRK1/2/3 fusion as a tumor-agnostic genomic alteration (ESCAT score IC) (35, 36). For other molecular targets with an agnostic ESCAT 1 score [MSI-H, RET fusions, BRAF mutations, FGFR1/2/3 fusions and mutations, and tumor mutational burden high (TMB-H)], AIFA approval is lacking, making it rather difficult to use these drugs (15, 16, 37). Thanks to the joint action of the ISG and patient associations, it has recently been possible to obtain from the Italian Medicines Agency (AIFA) the possibility of administering crizotinib under Law 648/1996 (and therefore covered by the National Health System despite not being approved for use in the specific pathology) in patients with inflammatory myofibroblastic tumors with ALK fusion (ESCAT score IB), as well as regorafenib in osteosarcomas and atezolizumab in alveolar sarcomas, based on clinical data on their clinical activity in specific histologic subtypes (15, 38). Beyond these specific drugs, any other druggable molecular alterations with ESCAT scores II and III become targets for which the use of specific drugs is difficult to achieve. This is often the case whether or not these alterations are evaluated for individual patients within the MTB and outside of clinical trials. The use of these drugs depends on the availability of AIFA funds or the possibility of use within the framework of expanded access to the drug, supported by pharmaceutical companies or procedures paid for by individual hospitals (off-label use of the drug) (39).

Overall, studies that have used NGS in sarcomas have shown that approximately half of the cases have clinically actionable mutations, although it remains unclear how many of these mutations are the primary drivers of the disease rather than secondary mutations acquired later in the development of the disease and how much, therefore, their targeting may have therapeutic significance (20, 40).

A report by Boddu et al, in which the tumor tissue of 114 sarcoma patients was analyzed in NGS, showed that 96.4% of them had mutations (17). This percentage was approximately 70% in a study conducted in a pediatric population including sarcoma patients, and 67%, with multiple alterations in nearly 70% of cases, according to data from the ProfiLER 01 study, which showed TP53, RB1, CDKN2A, CDK4, MDM2, and PTEN as the most frequently affected genes (41, 42). An analysis of the pediatric patients of the ProfiLER trial (including, 47% of sarcoma patients) and of the INFORM study, showed a druggable mutation rate of 23% and of 50%, respectively (40, 43). Actionable alterations were identified in 31.7% of cases, microsatellite instability (MSI) in less than 0.3% of cases and high tumor mutational burden (TMB) in only 3.9% of cases in a very large study by Gounder et al. (19). A low MSI and TMB were also confirmed by other reports on sarcomas (44, 45). Overall, about two-thirds of the molecular alterations identified were considered actionable in a real world study by Lopes-Brás et al, most of which had shown clinical benefit in other cancers or were under investigation in clinical trials (44). In the European Organisation for Research and Treatment of Cancer (EORTC) SPECTA-AYA study, aimed to analyze adolescent and young adult sarcoma patients, an indication for individualized treatment following molecular analysis was posed in 81% of eligible patients, but the recommendation was generally based on a weak level of evidence and on activity in other tumor types (46). In the experience of Regina Elena National Cancer Center, we identified actionable target in 57.9% of sarcoma patients, and 16% were treated with targeted therapy (20). Unfortunately, literature showed that only a small percentage of sarcoma patients with actionable target were then treated with target therapy (17, 41, 47). This is probably due to the paucity of literature data supporting a particular treatment, considering that most data in sarcomas derive from case reports or small case series, due to the aforementioned difficulty in conducting large clinical trials. In addition, the lack of supporting data also leads to difficulty in prescribing and accessing the appropriate treatment.

In this article we present the results of a survey, proposed in 2021 to referral centers for the treatment of sarcomas in Italy as part of the ACC’s MSKT, to assess the prevalence and organization of MTBs.

All the centers surveyed had an MTB and its composition was variable across centers, with a dedicated oncologist present in 83% of the cases. The survey showed that not all professionals were present at every meeting. For example, 83% of centers could rely on the support of the bioinformatician for molecular data analysis but was always present during meetings in only half of them. Similarly, the oncologist was essential for proposing new cases or discussing results in all the centers, but not all centers had a permanent oncologist at the MTB. Furthermore, the survey highlights the challenges in accessing adequate molecular techniques for identifying targeted therapies tailored to specific sarcoma histologic subtypes, as well as achieving uniformity in high-throughput panels and platform types.

Most centers complied with ACC guidelines for eligibility of patients to MTB evaluation and in most cases (83% of centers) molecular testing was proposed when therapeutic lines were exhausted. This finding is quite expected and may be justified by the fact that it is difficult to get a drug out of indication when standard therapeutic lines are still available, especially in rare diseases such as sarcomas, where most are not derived from prospective controlled trials. However, what is the best time to do molecular analysis remains a point of debate for several reasons. Earlier analysis could indeed provide useful information about the disease and possible responsiveness to innovative treatments when the patient’s clinical condition is still good. In addition, starting an analysis earlier would shorten the time to access target drugs, given the often long time to get the results of molecular analysis and to evade the administrative procedures to access drugs that are often off label. It is also to be considered that there are sarcoma histologic subtypess that are known to be unresponsive to standard chemotherapy treatments and for which the anticipation of molecular analysis could be useful. Finally, one other setting in which molecular analysis might be anticipated is in the presence of suspected association with genetic diseases.

Regarding the material tested, metastatic tissue was analyzed in 100% of cases and combined with the primary tumor in 83% of centers. Sarcomas often undergo surgery for metastases, especially isolated lung metastases, so in many cases this type of biological tissue is already available at the time of MTB discussion, offering quite a unique opportunity to analyze potential druggable mutation relatively late in the clinical history and right before potential use of the information.

This survey shows that the time taken to obtain molecular analysis results varies from site to site. Two centers reported less than 10 days, which compares well to literature data, in which much longer times are noted, from about 40 days in a study by Sholler et al. to 7 weeks required by the MULTISARC protocol (48, 49).

While the responses from the different centers are fairly homogeneous regarding the analyses carried out, with most of the techniques available in all the centers, we noted a greater heterogeneity about the analysis software and evidence criteria for assessing the actionability of the targets used. From this point of view, greater cooperation between the centers to standardize the techniques used would be highly beneficial.

The survey demonstrates that most centers (83%) collected MTB results and therapeutic decisions in a report; data obtained, and follow-up of patients were stored in a database locally. This finding is important because cooperation between centers and centralized case collection could lead to faster progress in personalized medicine for the treatment of sarcomas through the publication of data from high-volume centers that would strengthen scientific evidence. For this purpose, the PROGENSARC study (GENomics PROphylation in SARComas for therapeutic purposes), a retrospective/prospective study aimed at collecting genomic profiling data in sarcomas, is currently ongoing in sarcoma referral centers in Italy.

Interestingly, a French randomized multicenter prospective study, the MULTISARC study (NCT03784014), is currently ongoing to evaluate the utility of NGS in clinical practice in patients with sarcoma. In this study, patients with pre-treated advanced STS are randomized in a 1:1 ratio to perform or not perform NGS before a further line of treatment (48). The primary objective is to assess whether NGS can be conducted for a large proportion of metastatic STS participants within a reasonable timeframe and, secondarily, to determine whether an NGS-guided therapeutic strategy improves participant’s outcome. Preliminary results on 439 patients were presented at the 2024 ESMO congress showing that 47% of patients exhibited a target mutation, and 36% received the experimental NGS-based treatment (50).

While genomic profiling for therapeutic purposes still has lights and shadows and has not yet entered in the clinical practice, the use of molecular methods at the diagnostic phase is now of routinary use in referral centers to confirm the histopathologic diagnosis in sarcomas characterized by specific molecular alterations (16). Gounder et al. observed a refinement or correction of the diagnosis in 10.5% of cases due to molecular analysis (19). A diagnosis change has also been observed in other studies, such as the GENSARC study, which observed a modification of diagnosis in 53 out of 384 patients (13.8%), concluding that molecular analysis should be a mainstay for the diagnostic process of sarcoma also in centers where the histological analysis is made by an expert pathologist (16–18, 46).

The main limitation of this study is the small number of centers that participated in the survey. One possible reason for the low response rate is that the approach was sporadic due to the absence of fully established MTBs at the time of the survey. The value of this study lies however in its provision of a descriptive analysis of the organization of certain specialist centers, both for adults and children, and the clinical need to carry out genetic profiling for these complex and heterogeneous conditions.Although the survey only provides a snapshot of the early stages of MTB use in patients with rare cancers, such as sarcomas, and is not fully representative of the national picture the results offer insights into the state of MTB organization at that specific time before widespread adoption of Comprehensive Genomic Profiling (CGP). It has moreover prompted a multicenter, retrospective collection of cases that have undergone genomic profiling for therapeutic purposes up to the most recent times (paper in submission). In conclusion, due to the rarity and complexity of sarcomas and the difficulty of applying the ESCAT score in the absence of sufficiently powered clinical studies, one of the ISG’s current missions is to collect prospective molecular data on various sarcoma histologic subtypesin a national registry/database. This will generate data-driven hypotheses and facilitate the development of new treatments, as well as the standardization and homogenization of molecular profiling procedures and MTB organization among centers (consensus paper recently accepted for publication). Ideally, an ESCAT score would also be implemented for rare tumors, alongside a virtual national MTB on sarcomas, in collaboration with the National Rare Tumors Network.

## Data Availability

The datasets presented in this study can be found in online repositories. The names of the repository/repositories and accession number(s) can be found below: The datasets during and/or analysed during the current study available from the corresponding author on reasonable request.
